# The Treatment of the Bone Deformity of the Thorax and Spine in Scoliosis by Plaster Jackets

**Published:** 1907-06

**Authors:** 


### THE TREATMENT OF THE BONE DEFORMITY OF THE THORAX AND SPINE IN SCOLIOSIS BY PLASTER JACKETS, UTILIZING THE EXPANSION OF THE LUNGS AS A CORRECTIVE FORCE.

Drs. Michael Hoke and Chas. Andrews report satisfactory results from the treatment of scoliosis by the method indicated in the above title and give a number of illustrations showing the marked improvement that may be obtained from its application. They give the following conclusion:

The result obtained by reversing under pressure the method of respiratory expansion in these cases of scoliotic deformity must force one to conclude that: The disturbance of the symmetrical expansion by its dynamic influence upon the ribs must be a factor in permitting the spine to bend and therefore be a constant influence in the rotation of the spine, as well as a most powerful factor in determining the shape of the deformed thorax associated with the socoliosis; that in the treatment of the condition these principles must be applied in the treatment of the bone deformity.

It is not intended in this article to minimize the other causative factors, anatomical, static, physical, etc., so long and correctly recognized as causative influences in the production of the deformity. Whatever causes may produce the scoliotic posture, increase the respiratory forces in a part of the chest, diminish them in other parts as explained in this article, so that this changed respiration adds to the rotation, forces the chest wall into a prominence where the respiration is exaggerated, and permits the chest to flatten where the respiration is diminished. The vicious circle is made complete by this alteration of the respiratory expansion.—New York Medical Journal, May 11th, 1907.

